# Hypertensive disorders of pregnancy: what the physician needs to know

**DOI:** 10.5830/CVJA-2016-051

**Published:** 2016

**Authors:** John Anthony, Albertino Damasceno, Dike Ojjii

**Affiliations:** Division of Obstetrics and Gynaecology, Groote Schuur Hospital, University of Cape Town, Cape Town, South Africa; Department of Cardiology, Faculty of Medicine, Eduardo Mondlane University, Maputo, Mozambique; Department of Cardiology, University of Abuja, Abuja, Nigeria

**Keywords:** valvular, heart disease, pregnancy

## Abstract

Hypertension developing during pregnancy may be caused by a variety of different pathophysiological mechanisms. The occurrence of proteinuric hypertension during the second half of pregnancy identifies a group of women whose hypertensive disorder is most likely to be caused by the pregnancy itself and for whom the risk of complications, including maternal mortality, is highest. Physicians identifying patients with hypertension in pregnancy need to discriminate between pre-eclampsia and other forms of hypertensive disease. Pre-eclamptic disease requires obstetric intervention before it will resolve and it must be managed in a multidisciplinary environment. The principles of diagnosis and management of these different entities are outlined in this review.

## Abstract

Hypertension during pregnancy is widespread, representing the most common medical complication of pregnancy and affecting 6–8% of gestations in the United States of America. Two hospital-based studies in sub-Saharan Africa have put the prevalence of this disorder at 11.5 and 26.5% of all deliveries, respectively.^1^,^2^ There are four categories of hypertension in pregnancy, chronic hypertension, gestational hypertension, pre-eclampsia, and pre-eclampsia superimposed on chronic hypertension, as defined by the National High Blood Pressure Education Program Working Group in Pregnancy.

Hypertension during pregnancy is not only common but also associated with a risk of morbidity and mortality.^3^,^4^ The risk of adverse outcomes during pregnancy is largely but not exclusively confined to those pregnant women diagnosed to have pre-eclampsia.^4^,^5^ The separation of hypertension during pregnancy into pre-eclampsia or non-pre-eclamptic disease is a foundational consideration when determining the likely course of the disease, the necessary management and the probable outcome.^3^

Pre-eclampsia is uniquely manifest during pregnancy and is associated with a pathophysiological phenotype that encompasses placental disease, growth restriction of the foetus and the development of severe but reversible hypertension during pregnancy.^4^,^6^,^7^ Chronic hypertension, regardless of the precise diagnosis, is not specifically associated with placental vascular disease or severe intra-uterine growth restriction and will not remit after delivery.^8^ The necessary level of surveillance, hospitalisation and the need for preterm delivery rests upon the distinction between these hypertensive diagnoses.^9^

In this review we discuss the different types of hypertension during pregnancy, and the physician evaluation, including physical examination and laboratory investigations of the hypertensive pregnant patient.

## Pre-eclampsia

## Epidemiology

Pre-eclampsia affects one in 30 primigravid women and one in 60 women in their second or subsequent pregnancies.^10^ Those who have suffered from the condition before are more likely to develop it in subsequent pregnancies (a one-in-seven risk) and women with underlying co-morbidity are also more likely to develop this complication of pregnancy. Specifically, women with chronic hypertension have a 25% risk of developing superimposed pre-eclampsia, and women with collagen vascular disease are also more prone to develop pre-eclampsia.^8^,^9^,^11^ There is also a hereditary component, and obesity is strongly associated with the risk of developing the condition.^12^

Obstetric risk factors include an increasing risk of developing pre-eclampsia related to multiple and even higher-order multiple pregnancies. A large placenta, such as those seen in women with trophoblastic disease or various kinds of foetal aneuploidy, are also associated with an increased risk of developing pre-eclampsia. Other risk factors that have been identified as leading to an increased probability of pre-eclampsia developing during pregnancy include antiphospholipid antibody syndrome, chronic hypertension, chronic renal disease, a maternal age over 40 years, nulliparity, incidence of pre-eclampsia in a previous pregnancy and pre-gestational diabetes.

The highest incidence of pre-eclampsia is among women having their first baby, whereas the greater prevalence of the disease is in multiparous pregnant women. The disease is described as a condition of primigravidity but it is also, to some extent, associated with primipaternity.^10^

## Clinical phenotype

Pre-eclampsia is a syndrome characterised by the development of hypertension and proteinuria in the latter part of pregnancy, which then remits after delivery.^3^ Pre-eclampsia is unlikely to be the cause of hypertension or proteinuria developing before the 20th week of pregnancy.

Hypertension is defined in different ways but the most widely accepted definition is the sustained elevation of diastolic blood pressure above 90 mmHg over a period of four hours. Proteinuria is similarly defined in different ways but dipstick proteinuria of 1+ or more merits further investigation. The 24-hour urinary excretion of protein greater than 300 mg is regarded as being pathological.

Pre-eclampsia may present in an asymptomatic form. It may also develop acutely or progress to a phase of illness in which multi-organ disease becomes evident.^13^ This may include the development of eclampsia, cerebrovascular haemorrhage leading to stroke, renal failure either in consequence of acute kidney injury or associated with a progressive decline in renal function, pulmonary oedema for a variety of reasons, liver injury in the form of the HELLP syndrome (haemolysis, elevated liver enzymes and low platelets) or obstetric haemorrhage caused by abruptio placentae (commonly associated with pre-eclampsia). Many of these complications of pre-eclampsia may be lifethreatening to the foetus and the pregnant woman.^14^-^16^

Characteristically, the delivery of the baby signals the onset of disease resolution, although the mother may continue to exhibit worsening disease for up to 24 hours after delivery. The hypertension associated with pre-eclampsia may take up to six weeks to resolve completely, even if the risk of fulminant disease abates within 24 hours of parturition.

## Pathology and pathophysiology

Pre-eclampsia is a disease of defective placentation.^6^ The vascular adaptation in the vessels supplying blood to the placenta show signs of inadequate dilatation as well as evidence of lumina pathology, similar to atherosclerosis. The placenta itself is usually small and infarcted to a greater extent than is usually seen in normal pregnancy.

The evolution of the clinical phenotype follows these pathophysiological events in the placental bed. The precise mechanisms are not fully elucidated but some combination of systemic immune activation in response to an increasing maternal circulatory burden of trophoblastic tissue released from the ischaemic placenta combines with components of oxidative stress and an imbalance in the production of angiogenic and anti-angiogenic factors to give rise to changes in systemic vascular endothelial function.^17^,^18^

The volume-overloaded circulation of normal pregnancy is offset by endothelial-dependent vasodilatation to such an extent that normal pregnancy is characterised by falling blood pressure, despite the volume overload.^19^ In pre-eclampsia, the endothelial mechanism is disrupted and hypertension based upon vasoconstriction ensues. The pattern of hypertension may evolve through stages where the increased systemic pressure may be partly based upon increased cardiac output, compensatory for the diminished perfusion of the placenta through narrow vessels in the placental bed.^20^ The later evolution of the disease is due to defective vasoregulation and vasoconstriction associated with loss of intravascular volume through leaky capillaries and the onset of multi-organ ischaemia.^21^-^25^

Specific organs show patterns of ischaemic change, and haemorrhage with or without oedema. These include the brain, kidneys, placenta and liver.^26^-^28^ In the brain, the oedema is seen in the watershed areas of perfusion of the occipital lobe and has been designated as ‘posterior reversible encephalopathy syndrome’.^29^ Large haemorrhages can arise from ruptured vessels, with consequent mass effects, including tonsillar herniation, leading to death. The liver shows periportal ischaemia and haemorrhage in women with the HELLP syndrome, whereas the kidneys show evidence of endotheliosis, associated in some cases with acute tubular and cortical ischaemic damage.^21^,^28^

The cardiovascular and pulmonary changes seen are those of pulmonary oedema in severe cases, usually without other overt signs of heart failure.^13^,^30^

## Risk of morbidity and mortality

There are two major causes of death among women with pre-eclampsia, cerebrovascular haemorrhage and pulmonary oedema, and each account for roughly half the number of deaths.^16^ Other rarer causes include the rupture of a subcapsular haematoma, which may complicate the HELLP syndrome.

Cerebrovascular haemorrhage is related to severe hypertension.^31^ The threshold above which this risk escalates is the mean arterial pressure above which the cerebral autoregulatory function fails. This is commonly considered to be 140 mmHg. It is unusual for women to develop such severe hypertension without associated seizure activity. The development of eclampsia leads to severe hypertension during seizure activity and it is the reason why the case fatality rate for eclampsia is cited as one in 50, whereas the overall case fatality rate of pre-eclampsia is set at one in 1 500.^14^,^32^ The prevention of eclampsia is as important as the treatment of severe hypertension.

Pulmonary oedema may develop for different reasons. The iatrogenic administration of excessive amounts of intravenous fluids may lead to an absolute increase in preload, resulting directly in interstitial pulmonary oedema.^13^,^22^ A very high systemic vascular resistance can also elevate the pulmonary capillary wedge pressure, leading to an increased risk of pulmonary oedema.^33^ The left ventricular function may also be abnormal and commonly demonstrates some degree of diastolic dysfunction, although left ventricular systolic dysfunction is unusual.^22^,^23^

The loss of protein in the urine may lower the colloid osmotic pressure and contribute to development of the generalised oedema so characteristic of pre-eclampsia, with similar effects on the lungs. Changes in capillary permeability and the lymphatic drainage of the lungs all modulate the risk of pulmonary oedema in women with variable changes in vascular resistance and ventricular function. Consequently, the precise mechanism of pulmonary oedema cannot be simply attributed to heart failure in this condition.

## Management principles

Pre-eclampsia is not a condition that can be managed adequately outside a hospital environment.4 The definitive management of pre-eclampsia is delivery.4 Once manifest, the condition tends to worsen and it is unusual for delivery to be delayed by more than 10 to 14 days once the patient develops symptoms or signs of the condition. Because the foetus is at risk of impaired growth and likely to deliver prematurely, management needs to take place in an obstetric unit with access to the best available level of paediatric care. Any improvement in neonatal outcome can only be secured by minimising the risks of prematurity. This is accomplished by delaying delivery for as long as the mother’s condition can be considered to be satisfactory.^34^,^35^

The development of symptoms, an uncontrollable spike in blood pressure or the evolution of defined organ dysfunction signal the onset of life-threatening disease, requiring that the focus of treatment shift from the neonatal outcome to protecting the interests of the mother. Delivery at this point is inevitable and the neonate will need to be cared for in the best available circumstances. The second means of improving perinatal outcome revolve around the use of corticosteroids, given to the mother. These accelerate the maturation of the foetal lungs and lessen the likelihood of neonatal intraventricular haemorrhage in the newborn.^36^ In addition to the necessity of effecting delivery by either induction of labour or caesarean section, the obstetrician plays a role in preventing complications.

The prevention of eclampsia is ensured by the use of magnesium sulphate, given as a continuous infusion or as intermittent intramuscular doses.^37^,^38^ This has been shown to be effective in reducing the risk of developing eclamptic seizures (and recurrent seizures) without adversely sedating the foetus. The mechanism of action is poorly understood and the use of magnesium sulphate needs to be weighed against potential risks. These include the development of toxicity, which is more common in women with renal failure. Toxicity leads to respiratory arrest, which can be reversed with intravenous calcium gluconate.

Women who are fitting should have their seizures aborted with intravenous benzodiazepines. Women who continue to fit despite treatment or those who are unable to protect their airway because of a low Glasgow coma scale need intubation and mechanical ventilation until the pregnancy is over and the mother’s condition shows signs of improvement.^13^,^39^

Proper management of severe hypertension is always a priority. Drugs used to lower the blood pressure are a variety of agents, including direct-acting vasodilators (hydrallazine, dihydrallazine), calcium channel blockers (nifedipine), alpha-and beta-blockers (labetalol), and combined arterial and venous vasodilators (nitroglycerine). Potent vasodilators such as sodium nitroprusside or diazoxide should not be used because they are associated with a risk of precipitous decline in blood pressure.

## Specific organ failure is managed according to specific protocols

Eclampsia requires attention to seizure control as outlined above. Recurrent seizures may only be controllable by continuous infusion of propofol or diazepam; this usually requires intubation and ventilation for up to 24 hours after delivery has been effected. The co-morbidity associated with seizures needs individual management (see below); specific screening and treatment of aspiration pneumonia is important. Any focal neurological signs merit neuro-radiological investigation to exclude haemorrhage and infarction. The differential diagnosis of seizure activity also merits consideration and may extend to other possible diagnoses, including metabolic causes for seizure activity, thrombotic thrombocytopaenic purpura, systemic lupus erythematosis, cerebral venous thrombosis, malaria and amniotic fluid embolus.^40^
Renal failure may be manifest on the basis of diminished preload together with peripheral, including renal, vasospasm. Acute renal injury may also cause oliguria and azotaemia. This is the consequence of ischaemia (due to pre-eclampsia or pre-eclampsia complicated by hypovolaemia caused by abruptio placentae) and haemoglobinuria. The principles of management are those of cautious intravascular volume expansion (no more than 300 ml of colloidal solution given as a bolus dose) and vasodilatation.^41^ Renal failure that fails to respond to these measures should result in a policy of fluid restriction, management of actual or incipient hyperkalaemia and expectant management in anticipation of gradual recovery after delivery.^42^ In the acute phase of the illness, dialysis may be necessary.
Liver injury is associated with the HELLP syndrome. This condition needs to be distinguished from other causes of micro-angiopathic haemolytic anaemia as well as other causes of liver failure. The differential diagnosis therefore includes thrombotic thrombocytopenic purpura, acute fatty liver of pregnancy, auto-immune disease, malaria and sepsis. The hallmark of the HELLP syndrome is that it reverses after delivery, with the nadir of thrombocytopaenia occurring on the third day postpartum.43 The management is obstetric, meaning delivery. Patients who do not exhibit the characteristic resolution of the thrombocytopaenia merit investigation for other causes of micro-angiopathic haemolytic anaemia. The only lethal complication of the HELLP syndrome is the development of a large subcapsular liver haematoma, which ruptures, causing massive intraperitoneal haemorrhage.^44^ The liver injury itself and the elevated liver enzymes seen in HELLP syndrome are not associated with failure of hepatic synthetic function and do not usually lead to coagulopathy or hypoglycaemia. These features, if present, indicate an alternative diagnosis.
Pulmonary oedema is the most difficult complication of severe pre-eclampsia in which to make a specific diagnosis.^30^ The mechanisms of pulmonary oedema are outlined above and the differential diagnosis will include other causes of acute dyspnoea, commonly infection and embolus. Pulmonary oedema itself may be the consequence of pre-eclampsia, or pre-eclampsia complicating underlying illness. These illnesses may include valvular heart disease and ventricular dysfunction due to cardiomyopathy. Regardless of the cause, emergency management is usually the same, involving supportive management of oxygenation and various combinations of diuretic and vasodilator therapy with a view to reducing both afterload and preload. This is commonly accomplished by using direct-acting vasodilators, such as dihydrallazine, together with intravenous furosemide. The development of pulmonary oedema is a signal for investigation by means of radiology, ECG and echocardiography to try to ascertain as closely as possible what the underlying cause may be. In some circumstances, the acute management of critically ill women may be facilitated by the use of pulmonary artery catheters to directly measure haemodynamic variables.^45^ Pulmonary oedema complicating pre-eclampsia is also an indication for immediate delivery, to begin reversing the underlying pathophysiology of pre-eclampsia.

## Postpartum management

Delivery of the pre-eclamptic pregnant woman will trigger reversal of the underlying disease. Generalised oedema begins to dissipate as the capillary leak reverses and the pregnancy preload is excreted. Commonly, 48 to 72 hours after delivery, the left ventricular preload may start to increase as the oedema resolves.^46^ This is an appropriate time to facilitate a diuresis. The hypertension itself may persist for up to six weeks after delivery, requiring management for this duration with diuretics and second-line agents. Whereas angiotensin converting enzyme (ACE) inhibitors are commonly used in non-pregnant hypertensives, often calcium channel blockers are more rapidly effective in these circumstances and are a good choice of treatment for the limited period for which they will be required.

One of the most important aspects of managing the postpartum pre-eclamptic is that of counselling. Pre-eclampsia has been shown to be a marker of long-term risk. Specifically, there is an association between hyperinsulinaemia, dyslipidaemia and the risk of pre-eclampsia. These underlying metabolic disorders are also risk factors for early onset vascular disease (both coronary artery and cerebrovascular disease).^47^ Consequently, women with early onset pre-eclampsia are at risk of vascular arterial disease in later life. Attention therefore needs to be paid to primary prevention of these conditions through regular screening, and treatment for metabolic disorders. The second long-term consequence of pre-eclampsia is that of an increased risk of renal failure.^48^ This risk correlates with the number of pre-eclamptic pregnancies a woman may have and indicates a need to pay attention to aspects of care in later life that may have a renal protective effect, specifically the early and adequate treatment of hypertension.

## Chronic hypertension in pregnancy

Chronic hypertension during pregnancy may be divided into two groups: uncomplicated chronic hypertension and chronic hypertension with superimposed pre-eclampsia. The latter group requires management according to the principles outlined above, whereas the former requires out-patient care, often with an altered approach to therapeutic intervention. Chronic hypertension is defined as blood pressure of 140/90 mmHg or more on two occasions before 20 weeks of gestation or persisting beyond 12 weeks after delivery

## Chronic hypertension with superimposed pre-eclampsia

The risk of developing superimposed pre-eclampsia is estimated to be between 10 and 25%.^49^ The possibility of decreasing this risk merits consideration. The development of pre-eclampsia cannot be averted by controlling blood pressure and there is no therapy that has any major impact on the risk of developing superimposed pre-eclampsia. However, there is some evidence that the use of low-dose aspirin, given as a daily dose of 57 to 81 mg of aspirin, may reduce the risk of pre-eclampsia developing in about 10% of women who are at risk of the disease.^50^ It is not clear why aspirin is effective, and initial theories related to altered prostanoid metabolism have been discounted, with more recent speculation focused on the possible interaction between aspirin and the production of pro-inflammatory cytokines.^51^ Aspirin given in this dose is safe and has no effect on the foetus. Despite the modest effect on the incidence of the disease, it remains recommended therapy in women who are at risk.

The second strategy used to reduce the occurrence of pre-eclampsia is based on the prophylactic administration of large doses of oral calcium. Meta-analysis of the studies conducted to date indicate that calcium administered in doses of up to one gram three times a day may significantly reduce the occurrence of pre-eclampsia and may also reduce the development of severe hypertension.^52^ The criticism of this data arises from the observation that the two single largest studies in the meta-analysis failed to reach statistical significance. Despite these reservations, calcium supplementation is widely accepted practice during pregnancy where there is a suspected risk of pre-eclampsia.

Interventions that are not of benefit in preventing pre-eclampsia include bedrest, the use of anti-oxidant vitamins and antihypertensive therapy itself.

Given the imperfect prophylactic measures aimed at preventing pre-eclampsia, care of pregnant women with chronic hypertension requires appropriate precautions to ensure that the development of superimposed disease is detected early in its development because of the attendant risks of foetal and maternal morbidity and mortality. Knowing who will develop superimposed pre-eclampsia before it becomes clinically manifest would be useful information.

The clinical phenotype of pre-eclampsia arises from changes at the level of the foetoplacental unit and any early signs of intra-uterine growth restriction or abnormal uterine artery Doppler velocimetry may precede the onset of the clinical disease.^49^ The hallmark of superimposed pre-eclampsia is, however, the development of proteinuria. The difficulty with this is knowing when the proteinuria is a consequence of underlying pre-eclampsia rather than due to renal disease caused by longstanding hypertension or *a priori* renal disease with secondary hypertension (in many communities HIV-associated nephritis may be a major differential diagnosis).

This distinction may not be easily made on a clinical basis and where a diagnosis of pre-eclampsia enters the differential diagnosis, the patient deserves in-patient care and management for presumptive pre-eclampsia until an alternative diagnosis can be made. The natural history of pre-eclampsia sometimes facilitates the distinction between pre-eclampsia and renal disease as a cause for proteinuria because pre-eclampsia tends to worsen as the pregnancy continues, whereas the chronically hypertensive patient has an indolent condition that changes little with the passage of time.

The recent interest in biomarkers may provide an alternative way of diagnosing which hypertensive conditions have a placental origin. Angiogenic and anti-angiogenic factors [placental growth factor and the soluble receptor for vascular endothelial growth factor (sFlt)] have been shown to be good predictors of placental disease and may provide a ready means of discriminating between various types of hypertensive disease in pregnancy, specifically identifying those most at risk of adverse outcome.^53^

## Uncomplicated chronic hypertension

Uncomplicated chronic hypertension does not usually affect the pregnancy outcome to any significant degree. The drugs used to treat hypertension outside pregnancy may need to be revised and alternatives introduced in order to protect the foetus.

Physiological changes during pregnancy have an impact on chronically hypertensive women as well. Specifically, they will vasodilate during the second trimester, leading to a fall in blood pressure and a reduction in the requirement for treatment at this point in the pregnancy. As the volume expansion during pregnancy continues and peaks at about 32 weeks’ gestation, the need for treatment may increase again. The goals of treatment also may need to be revised during pregnancy.

Outside pregnancy, the aim of treatment is prevention of end-organ damage to the heart, vasculature and kidneys. The use of diuretics with ACE inhibitors is common and the goal of therapy is normotensive blood pressure. This strategy does not apply during pregnancy because the drugs may harm the foetus, and placental perfusion (in theory) may be adversely affected by antihypertensive drugs that diminish perfusion pressure.

Diuretics, although used to treat cardiac conditions during pregnancy, are generally held to be contra-indicated in the management of chronic hypertension during pregnancy because pregnancy relies upon volume expansion to secure an accelerated rate of delivery of oxygenated blood to the peripheral tissues, including the placental bed. ACE inhibitors are also contra-indicated because they may interfere with the physiological regulation of uterine blood flow through local uterine mechanisms. More seriously, they are associated with neonatal renal failure in children of women treated with them during pregnancy. Of the other categories of antihypertensive drugs, beta-blockers are also relatively contra-indicated, being considered to be an independent risk factor for the development of intra-uterine growth restriction.^54^

Antihypertensive therapy during pregnancy in chronically hypertensive women is usually secured through the use of alphamethyldopa or calcium channel blockers. The aim of treatment is to reduce the occurrence of severe hypertension to safer levels of blood pressure. Practically, the threshold for introducing treatment is a sustained increase in blood pressure to above 160/110 mmHg to levels below this without seeking to reduce the pressure to normotensive levels.

The complications of chronic hypertension during pregnancy may extend to various forms of cardiac decompensation, depending on the severity of the condition. Hence, hypertensive cardiomyopathy is rarely seen in relatively young women with chronic hypertension, although it may develop and can give rise to maternal mortality.^55^ More commonly, diastolic dysfunction caused by changes in left ventricular morphology may result in the onset of increasing dyspnoea in the third trimester as the volume expansion peaks out. Patients in this category are otherwise well, without any signs of superimposed pre-eclampsia. This is one circumstance where diuretic therapy may result in rapid clinical improvement and resolution of symptoms that will allow the pregnancy to continue to term.

Obstetric intervention is not commonly required in chronically hypertensive women. However, some mild degree of foetal growth restriction may be present and the risk of superimposed pre-eclampsia cannot be excluded with absolute certainty. Consequently, induction of labour is usually recommended for women who do not labour spontaneously before 40 weeks’ gestation.

## Latent hypertension

Pregnancy may render overt hypertension that is not yet clinically manifest outside of pregnancy. Women who have a strong familial history of hypertension, whose genetic predisposition will manifest as essential hypertension in later life, may become hypertensive during pregnancy. The mechanism is thought to be related to subnormal pregnancy vasodilatation in vessels, with a hereditary defect in vasoregulation. In this circumstance, the increased intravascular volume of pregnancy cannot be accommodated by adequate vasodilatation, with a rise in blood pressure developing in the late second to third trimester of pregnancy.^56^

This condition should be managed according to the same principles as those outlined for women with chronic hypertension. The outcome of the pregnancy is usually unaffected and the only consideration might be the need for induction of labour in women not yet delivered by 40 weeks’ gestation.

## Physiological hypertension

Hypertension does not always indicate disease. Pregnancy is characterised by massive plasma volume expansion, and the cardiovascular adaptation needed to accommodate this increased intravascular volume is that of equally massive peripheral vasodilatation. The net consequence of this is a fall in blood pressure during the second trimester, with increasing levels of blood pressure closer to term. The entire adaptation is mediated by the placenta, and the adequacy of the pregnant physiological change depends on the amount of biochemically active trophoblast in the uterus. Hence women with multiple pregnancies or those who have singleton pregnancies with a large placenta will have a greater degree of volume expansion than those with a smaller placental mass. The consequences of this may be a supraphysiological increase in plasma volume that exceeds the degree of compensatory vasodilatation close to term. These individuals have normal pregnancies in every respect, with normally grown babies and no other signs of pre-eclampsia. This is not a condition requiring treatment or intervention and should be recognised as a variant of normal.^3^

The difficulty of managing these patients lies in being certain that the distinction can be safely made between physiological hypertension and pre-eclampsia. For this reason, many of these women would be allowed to continue to term but induction of labour would be justified at 40 weeks’ gestation

## General evaluation of patients with hypertensive disorder of pregnancy

Determining whether high blood pressure identified during pregnancy is due to pre-eclampsia or chronic hypertension is sometimes a challenge to the physician, especially if there are no recorded blood pressures available from the first half of the gestation. Clinical characteristics obtained through a good history, physical examination and some laboratory investigations may be used to help clarify the diagnosis.

## Relevant history the physician must take

The time of detection of hypertension is very important. Hypertension occurring before 20 weeks’ gestation is almost always due to chronic hypertension, while new-onset hypertension after 20 weeks’ gestation should lead to a suspicion of gestational hypertension. Worsening hypertension after 20 weeks of gestation should lead to careful evaluation for the manifestations of pre-eclampsia.

Patients with pre-eclampsia may describe new-onset headache that is frontal, throbbing or similar to migraine headache. They may also have visual disturbances, including scintillations and scotoma, which has been linked to cerebral vasospasm. Gastrointestinal complaints, such as epigastric pain, may be moderate to severe in intensity and due to hepatic swelling and inflammation, with stretch of the liver capsule. Rapidly increasing or non-dependant oedema may be a symptom of developing pre-eclampsia. In addition, rapid weight gain as a result of oedema due to capillary leak, as well as renal sodium and fluid retention could be a pointer to pre-eclampsia. New-onset seizures in pregnancy suggest pre-eclampsia–eclampsia, but primary neurological disorders must always be excluded.

## Signs the physician must look out for

Pre-eclampsia is a multi-systemic disease with various physical signs. Oedema can be seen in non-dependent areas such as the face and hands, apart from the dependent areas. Maternal systolic blood pressure above 160 mmHg or diastolic blood pressure above 110 mmHg can occur and denote severe disease.

In measuring the blood pressure, women should be made to sit quietly for five to 10 minutes before each blood pressure measurement, and blood pressure should be measured in lateral recumbency with the cuff at the level of the heart. Korotokoff sounds I and V should be used to define the systolic and diastolic blood pressure, respectively. In about 5% of pregnant women, an exaggerated gap exists between the fourth and fifth Korotokoff sounds with the fifth sound approaching zero. In this type of case, the fourth sound may more closely approximate the true diastolic blood pressure.

Signs of secondary hypertension such as buffalo hump, wide purple abdominal striae suggesting glucocorticoid excess, systolic bruit heard over the abdomen or in the flanks suggesting renal artery stenosis, and radio-femoral delay or diminished pulses in the lower versus upper extremities suggesting aortic co-arctation should be looked for. The presence of a fourth heart sound on auscultation is not a normal finding in pregnancy and may suggest left ventricular hypertrophy from chronic hypertension. Carotid bruits may also reflect atherosclerotic disease due to longstanding hypertension. In addition, retinal changes of chronic hypertension may be noted. Retinal vasospasm and retinal oedema, which may manifest as severely impaired vision, generally reflects pre-eclampsia.

In pre-eclampsia right upper-quadrant abdominal tenderness stemming from hepatic swelling and capsular stretch may be seen. Although brisk or hyperactive reflexes are common during pregnancy, clonus is a sign of neuromuscular irritability that usually reflects severe pre-eclampsia.

## Laboratory investigations the physician must order

Laboratory investigations to evaluate chronic hypertension include testing for target-organ damage, and to exclude secondary causes of hypertension and co-morbid factors. For chronic hypertension in the first trimester, it is very useful to obtain a full blood count, electrolyte, urea and creatinine levels, liver enzyme concentrations and testing for proteinuria. These serve as baseline values to be referred to later in the pregnancy if there is a concern regarding superimposed pre-eclampsia.

Serum lipids usually increase during pregnancy and therefore measurement should be deferred until the postpartum period. Also, the increase in endogenous corticosteroids levels during normal pregnancy makes it difficult to evaluate for secondary hypertension due to adrenal corticosteroid excess.

Useful blood tests when evaluating eclampsia and pre-eclampsia include urinalysis, a full blood count, serum electrolyte levels, urea and creatinine 24-hour urinary protein excretion, and serum uric acid, liver enzyme and bilirubin levels.

## Follow up

The long-term implications of having a pregnancy complicated by pre-eclampsia or hypertension have been highlighted above. It is important that pregnant women with hypertensive disease be given every opportunity to attend appropriate follow-up care in order to prevent long-term premature morbidity and mortality.
